# All-Soft-Tissue Meniscus Allograft Transplantation With Circumferential Suture Tape Augmentation to Mitigate Hoop Stress and Promote Centralization

**DOI:** 10.1016/j.eats.2024.102954

**Published:** 2024-03-27

**Authors:** Jarod A. Richards, Jacob T. Williamson, David R. Woodard, David N.M. Caborn

**Affiliations:** a Department of Orthopaedic Surgery, University of Louisville (UofL), Louisville, Kentucky, U.S.A.; b UofL Health Sports Medicine, Louisville, Kentucky, U.S.A.; c Department of Orthopaedic Surgery, Missouri Orthopaedic Institute, University of Missouri, Columbia, Missouri, U.S.A.

## Abstract

Meniscus allograft transplantation (MAT) is a technically challenging procedure. Bone plugs, slot techniques, and all-soft-tissue fixation techniques have been described in the past. Each technique comes with advantages and disadvantages. Native menisci have circumferential collagen fibers to help resist hoop stress during loading cycles. Although hoop stress resistance is a known function of the menisci, its recreation in MAT has only been targeted indirectly through anatomic root placement. The authors describe the use of a high-tensile suture tape (i.e. InternalBrace) to promote centralization by directly mitigating hoop stresses through recreation of peripheral meniscus tensioning in MAT.

Meniscus allograft transplantation (MAT) is a technically challenging procedure that provides chondroprotective effects in patients with meniscus-deficient knees.^1^^,^^2^ MAT has demonstrated reproducible improvements in both patient pain and function.^2^, ^3^, ^4^ Graft survivability, however, is a persistent challenge plaguing arthroscopists globally.^5^ Failure rates are estimated to be approximately 30% at 10 years and 40% at 15 years.^5^ Despite decades of experience with and research into MAT technique, immense heterogeneity exists among surgeons in indications, graft choice, graft preparation, and fixation method.^2^^,^^5^

Surgeons typically describe their MAT fixation strategy as belonging to 1 of 3 groups: soft-tissue fixation, bone plug fixation, or bony slot fixation.^2^^,^^6^ Each root-fixation strategy has advantages and disadvantages. Bony fixation has greater biomechanical strength at time zero but is more technically demanding, has a greater risk of iatrogenic articular cartilage injury, and graft positioning can be restrictive due to bony attachments.^2^^,^^4^ Inversely, all-soft-tissue root fixation provides easier graft positioning but sacrifices bony stability and ingrowth potential.^2^^,^^4^ Some authors also purport bony fixation techniques allow for more anatomic root placement.^7^ Little Level I or II evidence exists to demonstrate graft fixation superiority, and meta and systematic analyses of lower levels of evidence do not elucidate improved graft survivability in either fixation type.^2^^,^^4^

Graft extrusion has been postulated as having the strongest influence on the chondroprotective ability of MAT due to an extruded graft’s inability to withstand hoop stresses.^1^^,^^8^ Centralization has emerged as a technique to mitigate meniscal extrusion in the setting of both meniscal root tears and MATs.^9^, ^10^, ^11^, ^12^ Several methods of centralization have been described, including arthroscopically placed suture anchors atop the tibial plateau and meniscotibial ligament reconstruction performed through a mini-open approach at the proximal tibia.^9^^,^^11^^,^^12^ The authors agree that anatomic root placement alone (regardless of fixation type) will not solve the problem of MAT extrusion. Arthroscopists must increase focus on the peripheral recreation of hoop stress resistance. Further advances in suture technology have provided arthroscopists with new solutions. The authors describe an alternative technique for MAT centralization using an all-soft-tissue allograft with peripheral, circumferential suture tape incorporation to recreate hoop stress resistance. The senior author has used this technique for both medial and lateral MATs.

## Surgical Technique (With Video Illustration)

### Graft Prep

**Fig 1:**
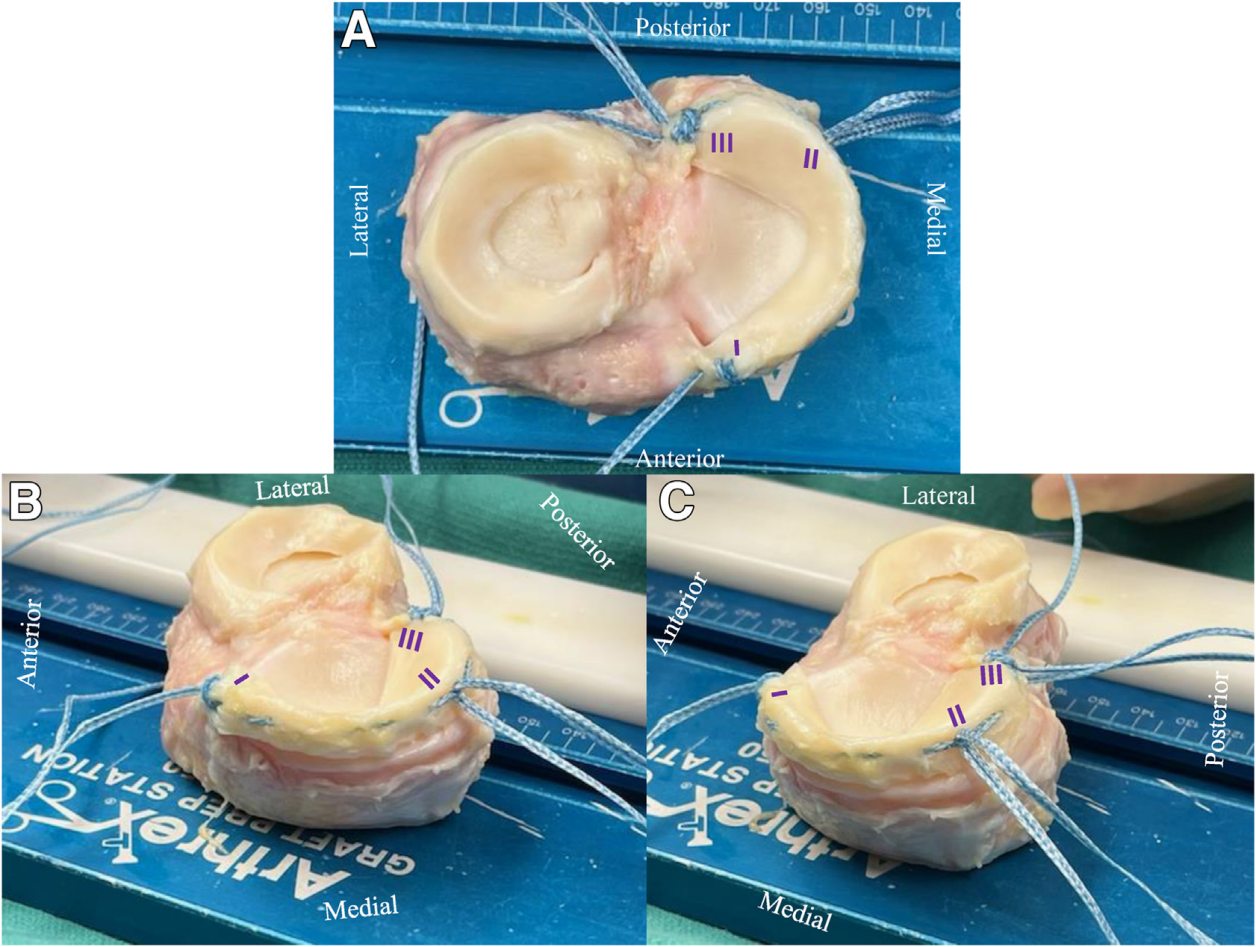
(a-c) Right proximal tibia allograft with medial meniscal preparation before meniscal amputation from the graft. Three points of fixation coincide with a peripherally coursing InternalBrace. The first suture tape is at the anterior horn with one exiting limb for fixation (I), the second at the posterior one-third junction of the meniscus with 2 exiting limbs for fixation (II), and the third at the posterior horn with three limbs exiting limbs for fixation (III). Note the second point of fixation (II) with 2 exiting limbs of suture tape wrapped around the InternalBrace at the posterior one-third junction of the medial meniscus has the capacity for approximately 1.5 to 2 cm of translation for appropriate meniscal seating once inside the joint.

An age-limited and size-matched fresh-viable proximal tibia osteochondral allograft is sourced from a tissue bank (MTF Biologics, Edison, NJ). Three total 2- mm FiberTape (Arthrex, Naples, FL) suture tapes are used, but any commercially available flat-braided suture of equivalent size, strength, and load-sharing capabilities^13^ would be suitable. The first suture tape is used to create a modified Mason–Allen stitch^14^ at the apex of the anterior horn of the meniscus (Fig 1). One limb of the first tape is left exiting the apex of the anterior horn. The other limb of the first suture tape is coursed through the meniscus periphery to create an InternalBrace (Arthrex) construct. This other limb of the first suture tape exits the apex of the posterior horn. A second suture tape is looped around the now formed peripheral InternalBrace (i.e., first suture tape as it courses through periphery) at the posterior one-third junction of the meniscus. Lastly, a third suture tape is used to create another modified Mason–Allen stitch at the posterior horn.^14^ Note that a peripheral, circumferential InternalBrace with 3 points of suture tape fixation has now been created (Fig 1). The first suture tape is at the anterior horn with one exiting limb for fixation, the second at the posterior one-third junction of the meniscus with 2 exiting limbs for fixation, and the third at the posterior horn with 3 limbs exiting limbs for fixation.

The second point of fixation with 2 exiting limbs of suture tape wrapped around the InternalBrace at the posterior one-third junction of the meniscus has the capacity for approximately 1.5 to 2 cm of translation. This is a critical element, as it allows for proper graft positioning once introduced into the joint (Table 1). A static point of fixation at this site could cause graft bunching should its drill tunnel not perfectly align. This point of fixation’s initial translation before tensioning allows for appropriate seating of the graft and subsequently recreation of hoop stress tension throughout the periphery of the meniscus via the InternalBrace. Furthermore, each of the 3 points of fixation (anterior horn, posterior one-third junction, and posterior horn) allow independent tensioning of the InternalBrace construct under direct arthroscopic visualization.

**Table 1:** Pearls and Pitfalls for Meniscus Allograft Transplantation With Suture Tape Augmentation

Pearls	Potential Pitfalls
• Tibial spine removal to assist with graft passage and introduction of marrow elements • Partial MCL release to assist with visualization if working medially • Posterior one-third junction suture with the ability for 1.5 to 2 cm of translation for proper graft seating within the joint	• Ensure a large enough bone bridge at the anteromedial tibia for independent posterior root and posterior one-third junction passage and tensioning • Lower-extremity alignment radiographs must be performed preoperatively to rule out concomitant deformity placing excess stress on the compartment

MCL, medial collateral ligament.

### Site Preparation

**Fig 2:**
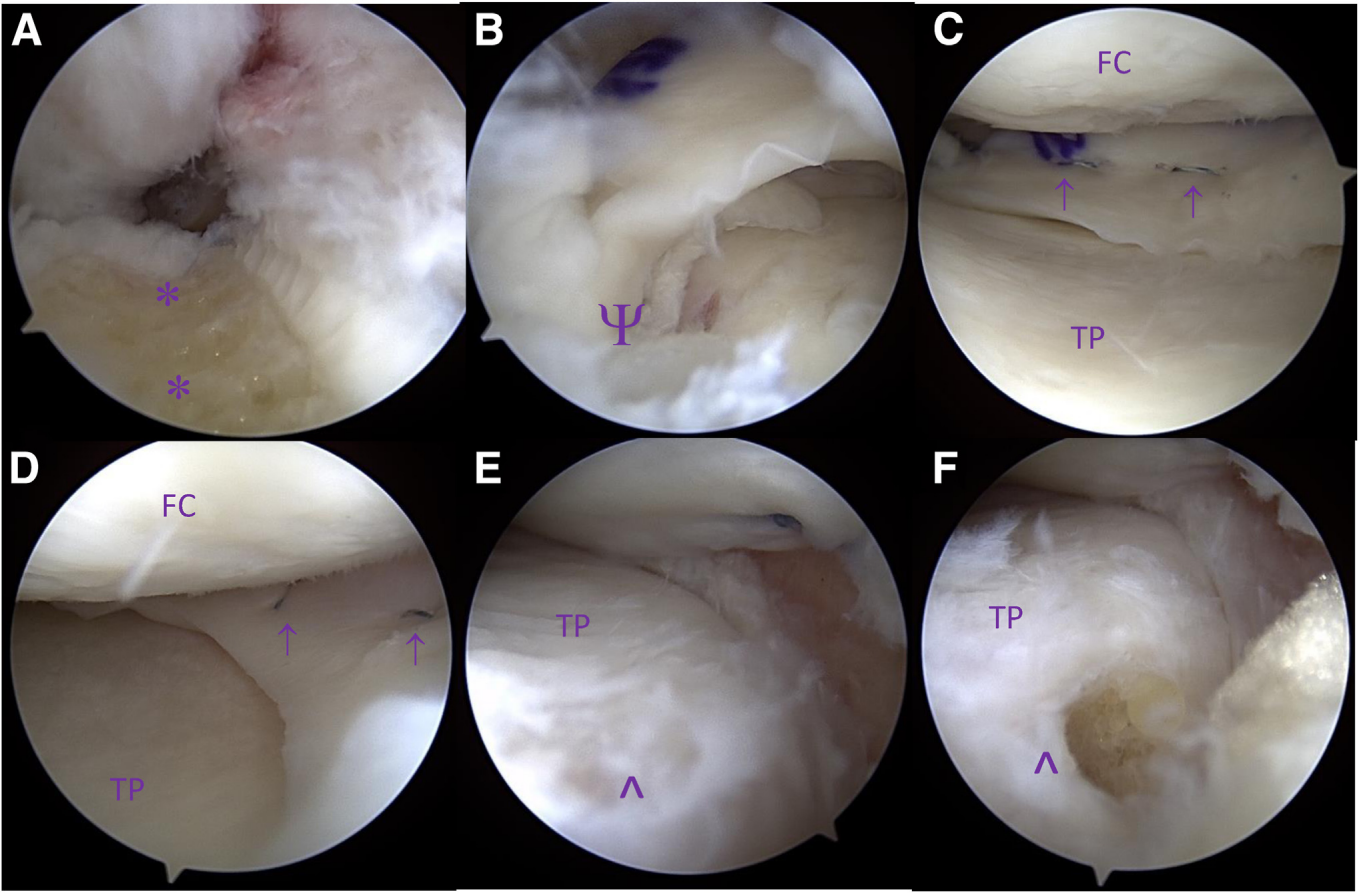
(a-f) Arthroscopic images taken at various points during the right medial meniscus allograft transplantation. (a) A view into the intra-articular notch demonstrating removal of the medial tibial spine (∗) to allow for better posterior horn visualization and the introduction of bone marrow contents. (b) The posterior horn of the medial meniscus allograft after introduction into its bone tunnel (Ψ). Note the native posterior horn remnant seated beneath the allograft root demonstrating anatomic placement of the graft. The remaining native tissue is subsequently debrided (not visualized). (c) The posterior one-third junction of the medial meniscus allograft with horizontal mattress sutures in place (↑). At this location, a second drill tunnel is placed before inside-out suture placement to accommodate the suture tape wrapping around the InternalBrace structure, which achieves circumferential tension. (d) The anterior one-third of the medial meniscus allograft with horizontal mattress sutures in place (↑). (e) Visualization of the native meniscus anterior root (^∧^). (f) Repeat visualization of the anterior root location (^∧^) with subsequent debridement of native meniscus and bone tapping before anchor placement demonstrating anatomic placement of the anterior root. (FC, femoral condyle; TP, tibial plateau.)

A standard diagnostic knee arthroscopy is performed. The meniscal remnant is debrided back to the level of the joint capsule. To assist with visualization, debridement of the posterior horn meniscal remnant, and later fixation of the posterior horn of the allograft meniscus, a few millimeters of the tibial spine can be removed with a burr with care taken to protect with anterior cruciate ligament and posterior cruciate ligament within the notch (Fig 2a). Should the medial compartment be tight and challenging to visualize, partial medial collateral release via a percutaneous technique can be performed to open the joint space and improve access with little to no long-term morbidity (Table 1).^15^^,^^16^

### Graft Introduction and Fixation

**Fig 3:**
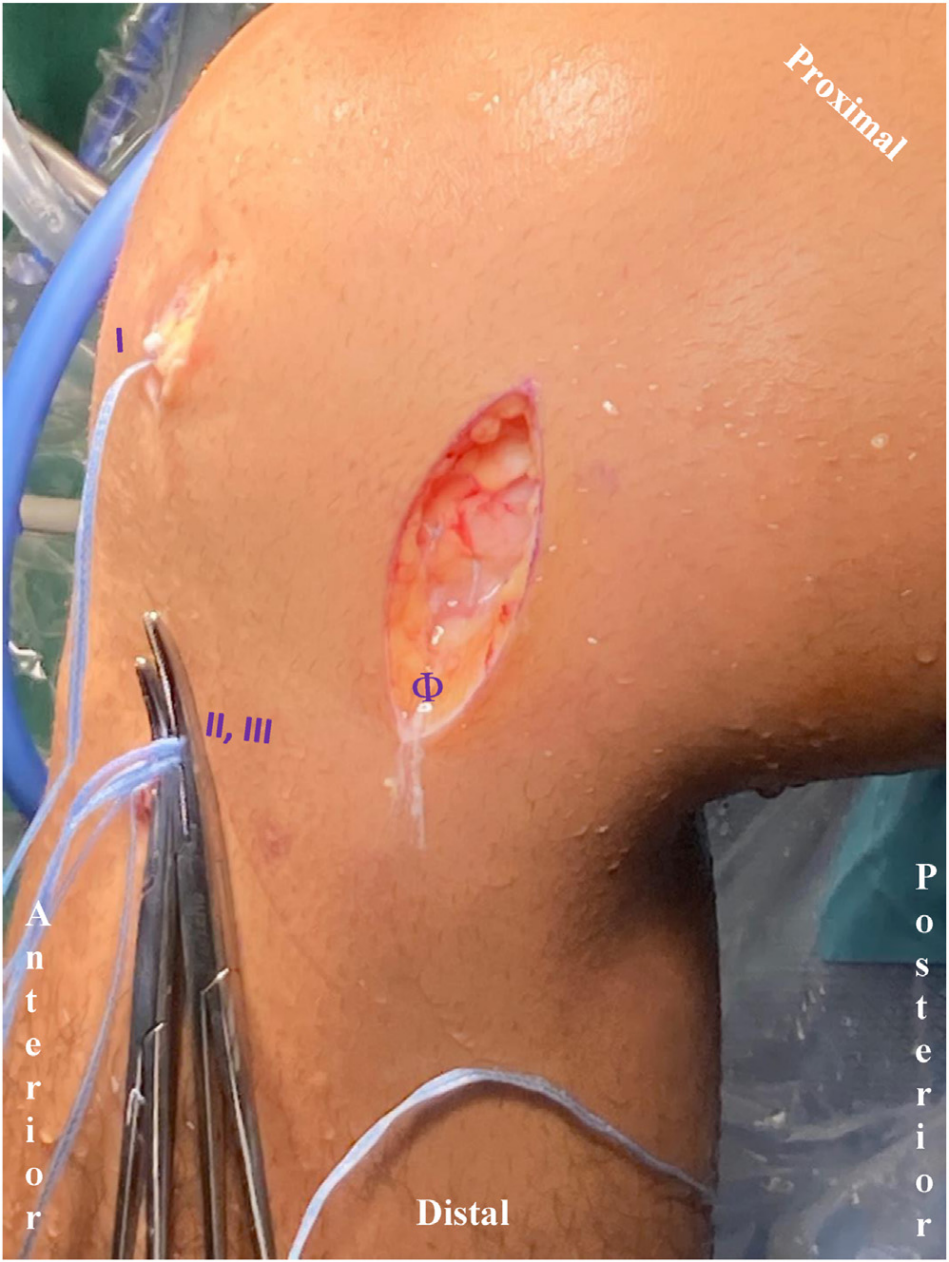
Medial aspect of the right knee. Two hemostats are used to provide tension on the posterior horn and posterior one-third junction suture tapes coming through their anteromedial drill tunnels (II, III). These will be tied over a bone bridge for final fixation. More posteriorly, a medial approach to the tibiofemoral joint line is used for an inside-out suture technique (Φ). Anteriorly, the anterior horn suture tape is exiting the medial arthroscopic portal prior to fixation with a PEEK anchor at the anterior root (I).

Care is taken to identify the posterior root attachment site on the tibia during remnant debridement. A drill guide is used in conjunction with a 6-mm FlipCutter (Arthrex) to create a 15-mm socket in the tibia at the site of the posterior root attachment. Passing sutures are introduced through the tunnel and clipped aside with a hemostat as they exit the anteromedial tibia for later use (Fig 3). Attention is then turned to the posterior one-third junction fixation site. A drill guide is set just past the chondral surface, this time in conjunction with a FlipCutter that is not activated to its wider setting (i.e., 3.5-mm nondeployed diameter) as this site will be used for passing sutures only and not the introduction of tissue. Passing sutures are introduced through this second tunnel and clipped with a hemostat as they exit the anteromedial tibia for later use (Fig 3). Note that a 10-mm bone bridge is maintained between the 2 bone tunnels exiting at the anteromedial tibia through a single skin incision.

The anteromedial arthroscopy portal is extended a few millimeters to allow introduction of the prepared allograft meniscus into the joint space. First, the posterior root passing sutures are used to shuttle the posterior horn of the meniscus (with three exiting suture tapes) into position. Posterior root meniscal tissue is then dunked into its 6-mm by 15-mm tunnel (Fig 2b). Any remaining native meniscus blocking this introduction should be debrided further. The 3 limbs of suture tape now exiting the anteromedial tibia are clipped at the skin with a hemostat under tension (Fig 3). The posterior one-third junction passing sutures are then used to advance the 2 free limbs of suture tape (which are wrapped around the InternalBrace to achieve circumferential tension) at the posterior one-third junction site of the meniscus allograft (Fig 2c). These 2 limbs of suture tape also exit the anteromedial tibia and are clipped with a hemostat under tension (Fig 3). Throughout this time, a blunt introducer can be used to help manipulate the meniscal allograft into the correct position. Once satisfactory positioning of the allograft meniscus is achieved and the posterior root has been provisionally seated within its bone tunnel, inside-out meniscus sutures are applied sequentially from the posterior to anterior direction in standard fashion as previously described (Fig 2c and d).^17^ Approximately 3 to 4 inside-out sutures are applied in both horizontal and vertical mattress technique as needed.

Attention is then turned to the anterior horn of the meniscus. Once the anterior root attachment site has been identified (Fig 3e), an awl followed by a tap is used to create a socket (Fig 3e) for a 4.75-mm PEEK (polyether ether ketone) SwiveLock anchor (Arthrex). The single limb of suture tape exiting the anterior meniscus is introduced through the anchor before it is advanced to provide anterior horn fixation. Afterwards, 2 to 3 inside-out sutures are applied in a similar fashion as previously described to achieve fixation of the anterior half of allograft meniscus. Lastly, the posterior horn sutures and posterior one-third junction sutures are final tensioned under direct arthroscopic visualization and tied over the aforementioned bone bridge. Alternatively, buttons or suspensory fixation could be used with the potential advantage for repeated tensioning.

## Discussion

The aforementioned MAT technique uses all-soft-tissue fixation to provide increased emphasis on peripheral hoop stress resistance recreation in the form of a suture tape construct (i.e., InternalBrace). The InternalBrace construct could theoretically be added to a bone plug or slot root fixation technique should a surgeon prefer bony root fixation. The authors contend that centralization through recreation of hoop stress resistance is of utmost importance. Anatomic root placement can assist with hoop stress resistance by appropriately tensioning circumferential collagen fibers, but further weight should be placed on peripheral hoop stress resistance recreation.

Extrusion has been cited as the main reason for all-soft-tissue fixation inferiority.^18^ A certain degree of physiologic extrusion is expected of the meniscus during the gait cycle, but the degree to which extrusion becomes pathologic is yet to be determined.^18^ Extrusion rates have been reported as approximately 40% in both all-soft-tissue and bony fixation MAT fixation techniques, with some studies reporting greater extrusion rates in all-soft-tissue cohorts and others reporting higher rates in bony fixation cohorts.^2^^,^^18^ It may be possible that root fixation has garnered too much attention, and more focus should be provided to peripheral meniscus tensioning to avoid extrusion.

Resistance to hoop stress is frequently cited as a basic biomechanical principle of the meniscus.^7^^,^^8^^,^^19^ Centralization is a means by which surgeons attempt to counteract hoop stress and prevent extrusion. Works by Krych and Smith have advanced the concept of centralization in both meniscal repair and MAT settings.^9^^,^^10^ Several techniques exist to achieve centralization (intra-articular suture anchor, mini-open meniscotibial ligament reconstruction).^11^^,^^20^ Each shares the common goal of providing additional fixation at the proximal tibia. The authors agree that the addition of a third point of fixation at the proximal tibia is an important concept, regardless of how it is achieved. The addition of a third fixation site in MAT is not a new concept. Stone et al.^21^^,^^22^ described a 3-tunnel technique in 1993 with modifications in 2003. Teo et al.^23^ recently expanded upon this with modern instrumentation. Although our described technique uses 2 tunnels and an anchor, it still provides 3 points of fixation, each attached to the InternalBrace.

Two distinct features of this technique promote centralization: a third point of fixation to the proximal tibia (in this case a drill tunnel at the articular margin of the posterior one third junction of the meniscus) and a load-sharing^13^ 2-mm FiberTape placed as an InternalBrace at the periphery of the meniscus. Advantages of this technique include ease of graft preparation and passage given the lack of bone plugs, the introduction of a high-tensile suture tape to mitigate hoop stresses, and 3 points of independently tensionable fixation under direct arthroscopic visualization (Table 2). Independent tensioning prevents overtightening and subsequent bunching at variable locations in the allograft. Disadvantages include the cost of added suture tapes, a lack of robust supportive biomechanical and clinical data, and the potential need to remove excess suture material in a revision transplantation scenario (not yet encountered by the authors).

**Table 2:** Advantages and Disadvantages of Meniscus Allograft Transplantation With Suture Tape Augmentation

Advantages	Disadvantages
• Ease of graft preparation and passage • High-tensile suture tape to mitigate hoop stresses • Three points of fixation for secure and independent tensioning/fixation	• Cost of added suture tapes • Further research needed for long-term outcome analysis

Further studies are needed to determine the efficacy of technique. Although the senior author has treated 6 individuals in this manner with no need for conversion to arthroplasty or MAT revision, the earliest with 5 years of follow up, the authors are aware of no objective outcome studies evaluating differences between all-soft-tissue MAT fixation techniques with or without InternalBrace suture application. Ideally, biomechanical studies with pressure mapping techniques similar to those employed by Ambra et al.^24^ and Wang et al.^25^ will be performed with a MAT InternalBrace cohort in the future. As new technology enters the market to both measure and recreate anatomic meniscal tensile forces, arthroscopists can continue to improve upon MAT survivability.

## Disclosures

The authors declare the following financial interests/personal relationships which may be considered as potential competing interests: J.A.R. serves on the editorial board for *Arthroscopy*. D.N.M.C. reports article publishing charges were provided by Arthrex and reports a relationship with Arthrex that includes: consulting or advisory. All other authors (J.T.W., D.R.W.) declare that they have no known competing financial interests or personal relationships that could have appeared to influence the work reported in this paper.

## Supplementary Data

ICMJE author disclosure forms

ICMJE author disclosure forms

ICMJE author disclosure forms

ICMJE author disclosure forms

Video 1 The accompanying video represents a technique for right medial meniscus allograft meniscal transplantation using suture tape augmentation as an InternalBrace. This technique highlights graft preparation, introduction, and fixation. This technique could be applied to medial or lateral meniscus transplantations.
