# Supplementary material for: All-Soft-Tissue Meniscus Allograft Transplantation With Circumferential Suture Tape Augmentation to Mitigate Hoop Stress and Promote Centralization
Source: Arthrosc Tech. 2024 Mar 27;13(5):102954. doi: 10.1016/j.eats.2024.102954 (PMC11144943; doi:10.1016/j.eats.2024.102954)
Supplement: ICMJE author disclosure forms [file mmc3.docx]

**Declaration of interests**
 
☐ The authors declare that they have no known competing financial interests or personal relationships that could have appeared to influence the work reported in this paper.
 
☒ The authors declare the following financial interests/personal relationships which may be considered as potential competing interests:

| Jarod Richards serves on the editorial board for Arthroscopy. |
| --- |
